# Tetracalcium Phosphate Biocement Hardened with a Mixture of Phytic Acid–Phytase in the Healing Process of Osteochondral Defects in Sheep

**DOI:** 10.3390/ijms242115690

**Published:** 2023-10-28

**Authors:** Maros Varga, Lenka Kresakova, Jan Danko, Katarina Vdoviakova, Filip Humenik, Pavol Rusnak, Maria Giretova, Tatiana Spakovska, Zuzana Andrejcakova, Marian Kadasi, Marko Vrzgula, Zuzana Criepokova, Sonja Ivaskova, Filip Korim, Lubomir Medvecky

**Affiliations:** 1 Hospital AGEL Kosice-Saca, Lucna 57, 040 15 Kosice-Saca, Slovakia; maros.varga@nke.agel.sk (M.V.); pavol.rusnak@nke.agel.sk (P.R.); tatiana.spakovska@nke.agel.sk (T.S.); 2 Department of Morphological Disciplines, University of Veterinary Medicine and Pharmacy in Kosice, Komenskeho 73, 041 81 Kosice, Slovakia; jan.danko@uvlf.sk (J.D.); katarina.vdoviakova@uvlf.sk (K.V.); filip.humenik@uvlf.sk (F.H.); sonja.ivaskova@student.uvlf.sk (S.I.); filip.korim@student.uvlf.sk (F.K.); 3 Division of Functional and Hybrid Systems, Institute of Materials Research of SAS, Watsonova 47, 040 01 Kosice, Slovakia; mgiretova@saske.sk (M.G.); lmedvecky@saske.sk (L.M.); 4 Department of Biology and Physiology, University of Veterinary Medicine and Pharmacy in Kosice, Komenskeho 73, 041 81 Kosice, Slovakia; zuzana.andrejcakova@uvlf.sk; 5 Clinic of Ruminants, University of Veterinary Medicine and Pharmacy in Kosice, Komenskeho 73, 041 81 Kosice, Slovakia; marian.kadasi@uvlf.sk; 6 Department of Anatomy, Faculty of Medicine, Pavol Jozef Safarik University in Kosice, Trieda SNP 1, 040 11 Kosice, Slovakia; marko.vrzgula@upjs.sk; 7 Clinic of Horses, University of Veterinary Medicine and Pharmacy in Kosice, Komenskeho 73, 041 81 Kosice, Slovakia; zuzana.criepokova@uvlf.sk

**Keywords:** calcium phosphates, phytic acid, phytase, regeneration, cartilage, subchondral bone, sheep, models, animal

## Abstract

Hyaline articular cartilage has unique physiological, biological, and biomechanical properties with very limited self-healing ability, which makes the process of cartilage regeneration extremely difficult. Therefore, research is currently focused on finding new and potentially better treatment options. The main objective of this in vivo study was to evaluate a novel biocement CX consisting of tetracalcium phosphate–monetit biocement hardened with a phytic acid–phytase mixture for the regeneration of osteochondral defects in sheep. The results were compared with tetracalcium phosphate–monetit biocement with classic fast-setting cement systems and untreated defects. After 6 months, the animals were sacrificed, and the samples were evaluated using macroscopic and histologic methods as well as X-ray, CT, and MR-imaging techniques. In contrast to the formation of fibrous or fibrocartilaginous tissue on the untreated side, treatment with biocements resulted in the formation of tissue with a dominant hyaline cartilage structure, although fine fibres were present (*p* < 0.001). There were no signs of pathomorphological changes or inflammation. Continuous formation of subchondral bone and hyaline cartilage layers was present even though residual biocement was observed in the trabecular bone. We consider biocement CX to be highly biocompatible and suitable for the treatment of osteochondral defects.

## 1. Introduction

Articular cartilage has a minimal self-healing ability due to its avascular nature, a small amount of chondrocytes with low metabolic activity, and no direct access to local progenitor cells. For these reasons, untreated lesions predispose patients to the further loss of articular cartilage, which leads to the development of joint diseases, mostly to the development of osteoarthritis [1,2,3,4]. The incidence of articular cartilage damage in our society is increasing significantly due to gradual ageing and the increase in obesity [5]. The unique structure of cartilage, which changes its mechanical properties in response to loading and compression, is difficult to replace. An important part of current regenerative medicine strategies aimed at replacing damaged tissues or contributing to their healing is the use of various types of biomaterials. The production of a biomaterial with properties corresponding to natural hyaline cartilage is a substantial and demanding task [2]. Although intensively studied, current treatment strategies involving different clinical approaches have their limitations. Therefore, the process of regeneration of articular cartilage is still a subject of great interest.

Biocements based on calcium phosphates (CaPs) are promising for various clinical applications due to their excellent properties, including biocompatibility, bioactivity, osteoconductivity, osteoinductivity, moldability, injectability, solubility, and biodegradability [6,7,8]. CaPs have been used for bone regeneration for decades and show advanced effects on bone healing [9]. CaPs are described as one or a combination of several types of calcium phosphate powders that, when mixed with a liquid, form a paste capable of self-solidification and hardening in situ at the defect site [10]. Currently, CaPs are usually used to fill and heal bone defects [8]. They can stimulate new bone formation through the upregulation of Bone Morphogenetic Protein 2 and enhancement of osteogenic differentiation of bone-marrow-derived mesenchymal stem cells [11]. Despite of above facts, the excellent formation of hyaline cartilage as well as subchodral bone with good mutual integration with surrounding tissues was revealed after the treatment of artificial osteochondral defects in pigs with CaPs containing an amino acid mixture [12,13].

Great effort has been made to increase the biological effect of CaPs and improve the composition; handling; and mechanical properties, such as setting, injectability, degradability, and porosity; as well as the healing ability of CaPs [7,8]. Studies focus on improving mechanical strength, tensile strength, degradation rate, minimizing the foreign body reaction, or increasing the fracture toughness [6,8].

CaPs are often combined with other biomaterials to improve and control their properties. Cement’s properties are often enhanced with native polymers, e.g., chitin, chitosan, alginate, gelatin, cellulose, collagen, and, also, synthetic polymers, e.g., poly (lactic-coglycolic acid), polycaprolactone, poly (L-lactic acid), and polyethylene glycol [6,14]. The efficiency of cement is increased by the addition of various growth factors or encapsulating drugs [15,16]. One of the supplements tested mainly in in vitro studies is phytic acid, primarily used as a phosphorus precursor. Currently, phytic acid is receiving considerable attention in the field of biomedicine due to its various beneficial properties. 

Phytic acid (C_6_H_18_O_24_P_6_) is a biogenic compound with high biological potential, abundantly found in seeds, grains, legumes, and nuts [17,18]. It is involved in the storage of mineral elements, DNA metabolism, and RNA transport [19]. In scientific studies, there is a growing interest in the further evaluation and understanding of in vitro osteogenic processes in which phytic acid participates [18,20,21,22,23,24]. Several authors have demonstrated the key role of phytic acid on alkaline phosphatase activity and mineralization, the reduction of osteoclastogenesis through RANKL signalling, and the differentiation of stem cells into osteoblasts [20,21,23]. Phytic acid considerably decreases pH, resulting in a positive effect on the bioresorption activity of osteoclasts [25] and acts on osteoblasts by increasing the production of osteopontin. The antioxidant and osteogenic properties of phytic acid may play a significant role in bone-remodelling processes. Moreover, phytic acid intake was associated with an increase in bone mineral density [18]. Hydrolysates of phytic acid, e.g., the phospholipid component of cell membranes and lipoproteins, are involved in various signalling pathways that affect cell growth and metabolism [26,27]. Phytates derived from phytic acid and the inositol complex appear to be essential for the repair of bone defects and the prevention of bone mineral loss [28,29].

Fungal phytase is a specific enzyme which hydrolyses phytic acid to lower inositol phosphates with the release of free phosphates. Enzyme catalysis through added fungal or bacterial phytase presents the most efficient method of phytic acid dephosphorization [30,31,32]. It was observed that the positive effect of the addition of phytic acid to CaPs on the in vitro cytotoxicity and the stimulation of the osteogenic potential of cells, as well as the possibility of controlling the setting process of CaPs via enzyme hydrolysis of phytic acid with phytase, was demonstrated [30,33]. 

Our study focused on evaluating the effect of the tetracalcium phosphate–monetit biocement hardened with a phytic acid–phytase mixture (biocement CX) on the healing of osteochondral defects. This in vivo study was conducted on the large animal model of sheep and the osteochondral defects were created in the trochlea ossis femoris. We expected that biocement CX applied to osteochondral defects could induce and stimulate the regeneration of bone tissue, as well as hyaline cartilage. The results were compared with biocement C composed of tetracalcium phosphate–monetit without the addition of phytic acid–phytase, with classic fast-setting cement systems with 2% NaH_2_PO_4_, and with untreated defects.

## 2. Results

### 2.1. General and Macroscopic Observations

The general state of health, the behaviour of the animals, and their condition were not negatively affected by the surgery. No abnormalities were found regarding water and food intake. Mild lameness was observed in the first 3 days postoperatively with gradual improvement up to 10 days after surgery. The temperature of the body, respiration rate, and pulse rate met normal values. No infection or other pathological changes in the wound were detected.

**Figure 1:**
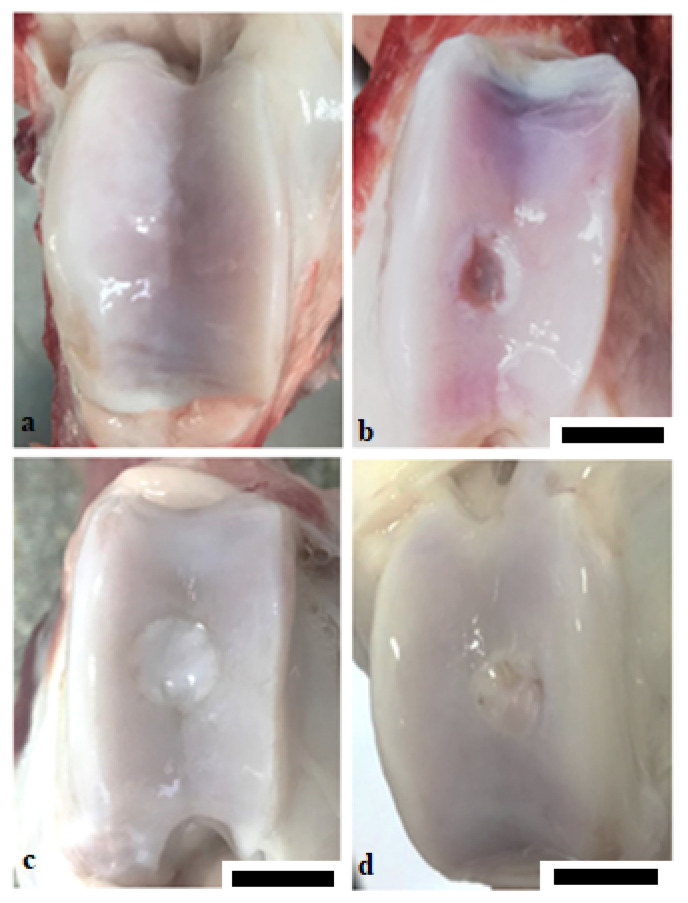
Macroscopic observation. Character of newly formed tissue 6 months after surgery. (**a**) control sample, native healthy cartilage of the trochlea ossis femoris in sheep; (**b**) untreated defect, large depression in the centre of the defect (**c**) osteochondral defect treated with biocement CX, defect surface covered with smooth, glistening tissue; (**d**) osteochondral defect treated with biocement C, visible slight surface irregularities; scale bar 1 cm.

On the treated sides, no free cartilage remains; Either osteophytes or biocement residues were found in the joint cavities. Synovial fluid was clear, normal in colour, and the usual amount. Macroscopically, the surgical lesions were evident in all cases, while the margins of neoformed tissue were firmly attached to the adjacent healthy hyaline cartilage. The colour of the neoformed tissue was whitish and slightly distinguishable from healthy natural cartilage with subtle surface irregularities. Osteochondral defects treated with biocement CX have a macroscopically better surface appearance and colour, as well as a more uniform structure and smoother surface, compared to defects treated with biocement C (Figure 1). Nevertheless, the differences in total scores between the two treated groups were not significant, according to the semiquantitative macroscopic scoring system. A significant difference was observed only in terms of surface homogeneity (*p* < 0.05), which showed better results after the CX application. Residues of both biocements were macroscopically detected in trabecular bone after the removal of osteochondral cylinders for histological examination.

**Scheme 1:**
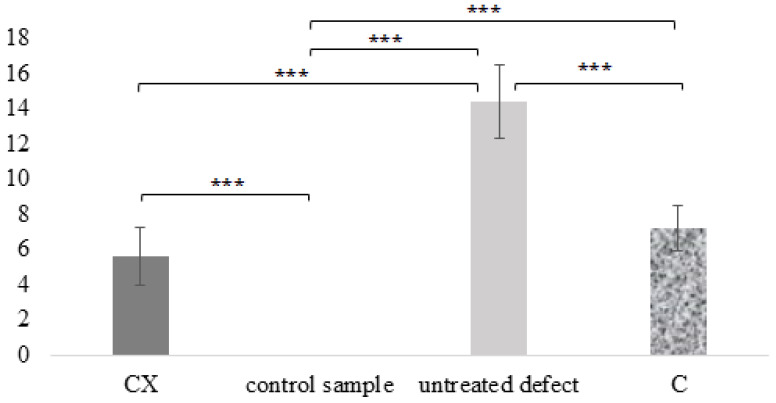
Results of the macroscopic semiquantitative scoring system. CX—osteochondral defects treated with biocement CX, control samples—healthy native cartilage obtained from osteochondral cylinders extracted by OATS kits; untreated defects—empty defects left to heal spontaneously C—osteochondral defects treated with biocement C, *** = *p* < 0.001.

Untreated osteochondral defects showed significantly smaller evidence of cartilage restoration and only partial repair with fibre-like, white or reddish tissue with cracks, deep depressions, and protrusions. In two cases, a larger amount of slightly coloured synovial fluid was noted in the joint cavity. Significant differences in total scores were observed between osteochondral defects treated with biocements and untreated defects (*p* < 0.001). The results of the total points number of the macroscopic semiquantitative scoring system are shown in Scheme 1 and Table 1.

### 2.2. Evaluation of Histological Samples

**Figure 2:**
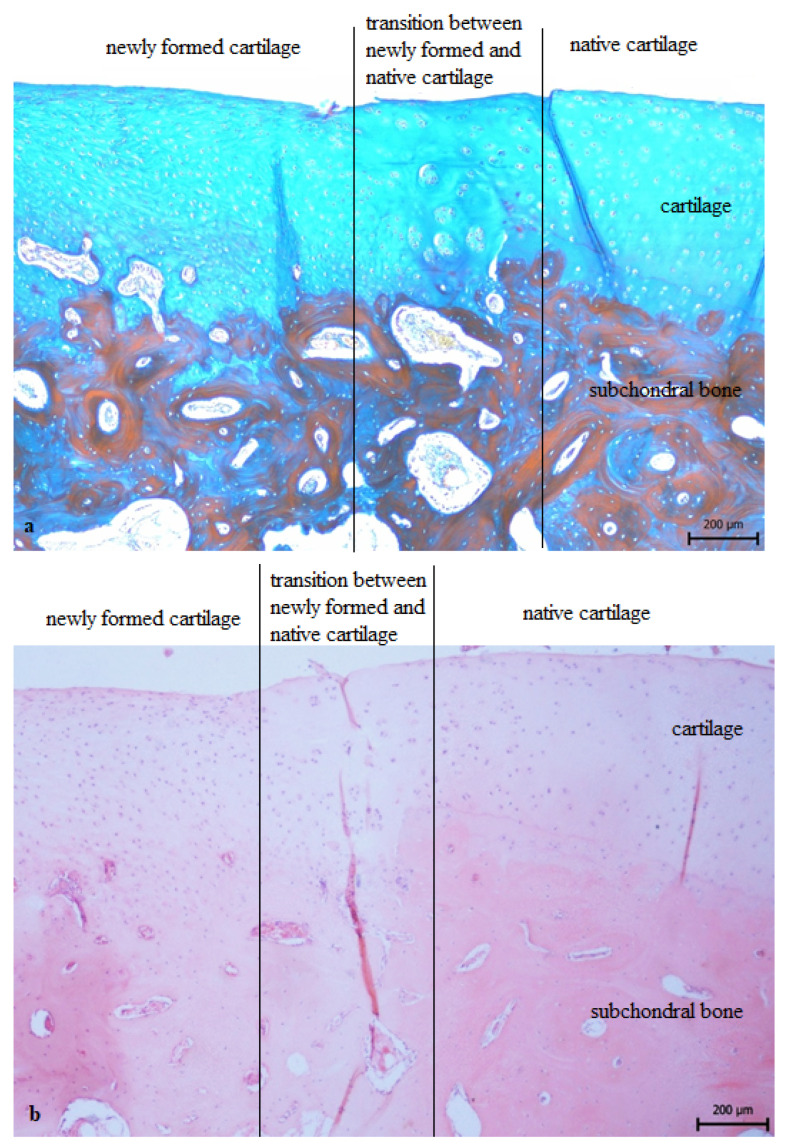
Histological evaluation of native cartilage, newly formed cartilage, and subchondral bone in sheep treated with biocement CX. Formation of continuous layers corresponding to hyaline cartilage with a moderate presence of fibres. Hypocellularity and clustering of chondrocytes were observed mostly at the transition between healthy and newly formed cartilage. (**a**) Masson’s trichrome staining, scale bar 200 µm; (**b**) Hematoxilin–eosin staining, 200 µm.

All osteochondral defects treated with biocement CX demonstrated no signs of degenerative pathological changes and showed the formation of continuous layers corresponding to hyaline cartilage in sheep. The histological structure of the newly formed tissue was predominantly hyaline, although a slight presence of fibres was observed in all cases. The tissue surface was smooth, slightly dented in places, and without fibrillation. The morphological character and arrangement of chondrocytes correspond to native cartilage, but mild hypocellularity and clustering of chondrocytes were observed in certain areas, especially at the transition between healthy and newly formed cartilage (Figure 2). Hypercellular segments were rarely monitored. The presence of cartilage tissue was verified by positive Safranin O and Alcian blue staining, indicating a significant amount of proteoglycans. The presence and homogeneous distribution of collagen were confirmed by Picrosirius red and Masson’s trichrome staining. Newly formed cartilage was firmly attached to the subchondral bone. The structure of the subchondral bone corresponds to the surrounding healthy subchondral bone.

**Figure 3:**
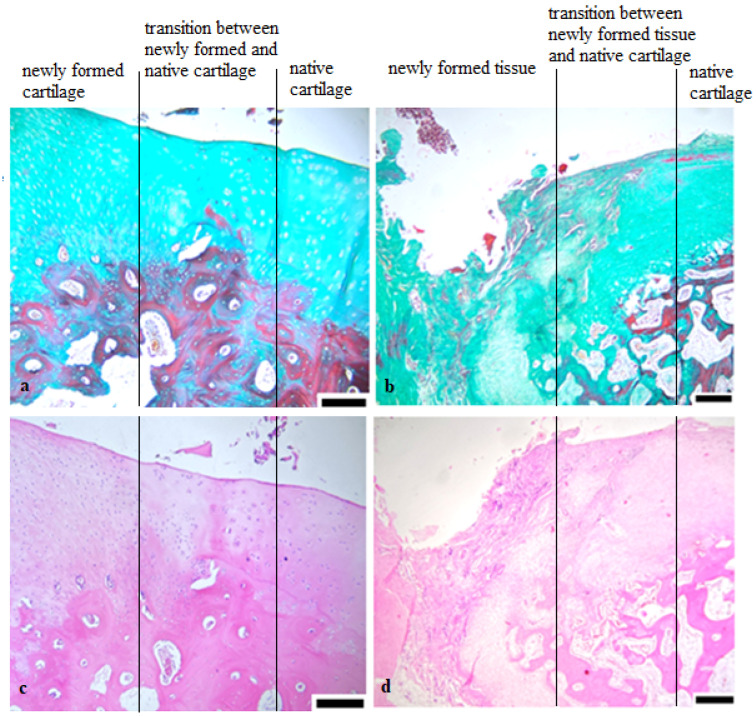
Histological evaluation. (**a**,**c**) Histological evaluation of newly formed tissue after treatment with biocement C. The organization of cartilage layers corresponds to native cartilage; fibrous fibres were also present. (**a**) Masson’s Trichrome staining, scale bar 200 µm; (**c**) Hematoxilin–eosin staining; (**b**,**d**) Untreated defect filled with mixed, disorganized tissue with a large depression, presence of vessel, fibrous tissue, and clefts; (**b**) Masson’s Trichrome staining, scale bar 200 µm; (**d**) Hematoxilin–eosin staining, scale bar 200 µm.

**Figure 4:**
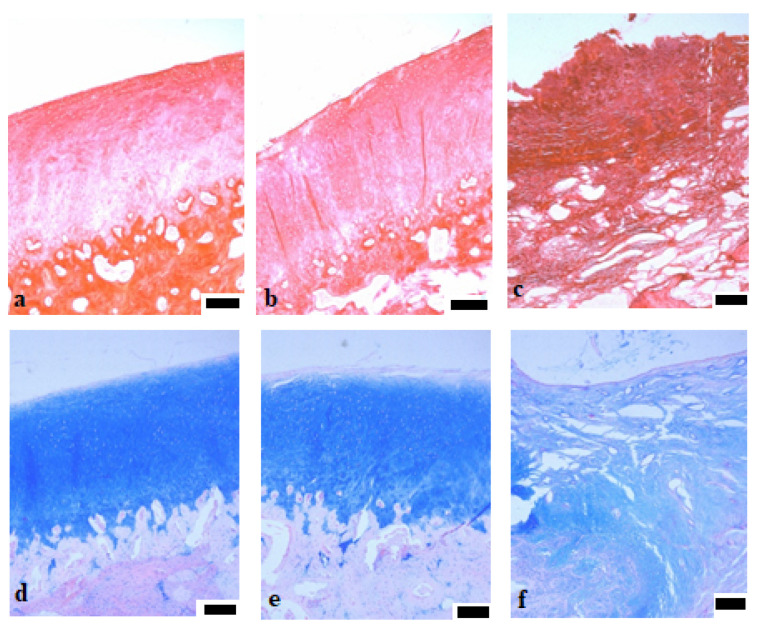
Histological evaluation. Homogenous tissue formation and distribution of collagen in samples treated with biocements; (**a**) sample of defect treated with biocement CX, picrosirius red, scale bar 200 µm; (**b**) sample of defect treated with biocement C, picrosirius red staining, scale bar 200 µm; (**c**) untreated defect filled with fibrous and disordered tissue. High presence of collagen fibres, picrosirius red staining, scale bar 200 µm; (**d**) Defect sample treated with biocement CX, proteoglycan deposition reaches a maximum in the middle zone. The content of proteoglycans in the tangential and radial zones was lower than in the middle zone. Alcian blue staining, 200 µm; (**e**) sample of defect treated with biocement C, the highest content of proteoglycans in the middle zone, Alcian blue staining, 200 µm; (**f**) untreated defect with low content of proteoglycans, Alcian blue staining, 200 µm.

In the group of animals treated with the biocement C, slightly smaller values were identified in the modified O’Driscoll scoring system compared to CX-treated defects. However, the differences between the CX and C groups in total score were not significant. We detected an irregular surface with depressions and cracks in three cases. In one case, superficial fibrillation was visible, and, similarly, in one case there was a layer of fibrous tissue with blood vessels on the surface of cartilage. The structure and organization of cartilage zones were similar to the defects that were treated with biocement CX. In two cases, we found an unclear formation of cartilaginous layers, and we noted both hypocellular and hypercellular areas in newly formed tissue. There were also regions with clusters of chondrocytes. The staining of Safranin O and Alcian blue was from medium to stronger intensity, which indicates the varying high content of proteoglycans. Positive staining of collagen with Picrosirius red and Masson’s Trichrome was observed (Figure 3 and Figure 4).

**Scheme 2:**
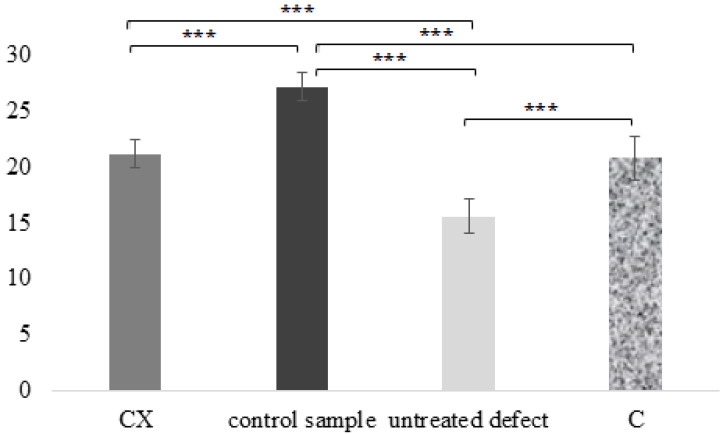
Results of modified O’Driscoll scoring system. CX—osteochondral defects treated with biocement CX, control samples—healthy native cartilage obtained from the osteochondral cylinders extracted by OATS kits; untreated defects—empty defects left to heal spontaneously. C—osteochondral defects treated with biocement C, *** = *p* < 0.001.

In contrast to treated defects, untreated defects left to heal spontaneously typically resulted in the formation of fibrocartilaginous tissue. In two animals, untreated defects were filled with fibrous, mixed, and disordered tissue (Figure 3 and Figure 4). The newly formed tissue in most cases showed surface irregularities, integrity, and homogeneity violations. In several cases, deep cracks and fissures extended to the calcified cartilage layer or to the entire depth of the defect. Compared to the treated defects, more fibrous tissue with vessels was present, as well as a higher incidence of acellular, hypocellular, and hypercellular zones. Clusters of chondrocytes were often found near fissures and areas filled with fibrous tissue. Similar to macroscopic evaluation, significant differences were observed between treated and untreated defects (*p* < 0.001) in total values, according to the modified O’Driscoll scoring system (Scheme 2).

### 2.3. X-ray and Computed Tomography Assessment

**Figure 5:**
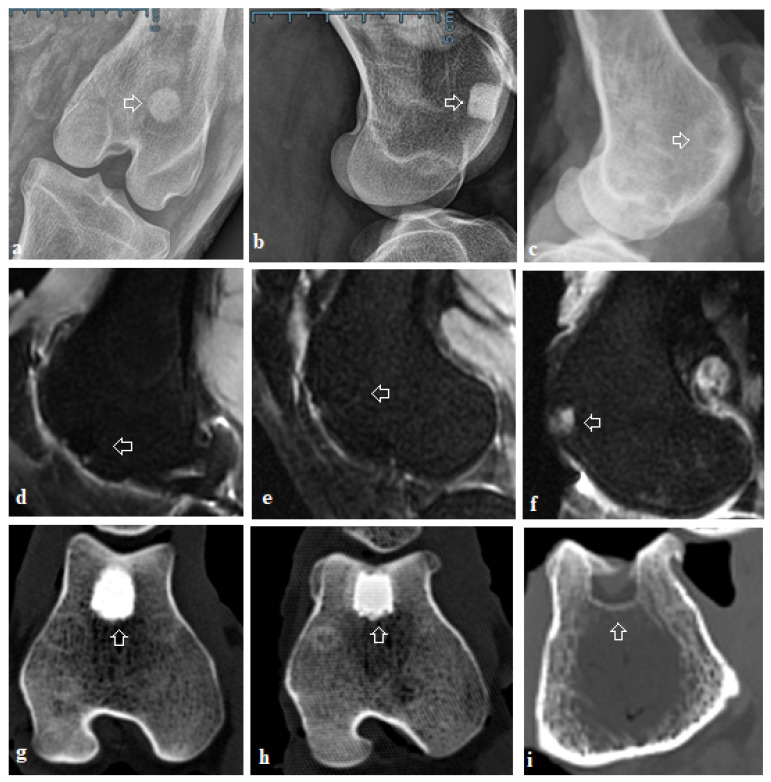
X-ray, CT, and MR imaging. (**a**) X-ray examination of the osteochondral defect after treatment with biocement CX; in trabecular bone, the unresorbed biocement was firmly attached to adjacent bone tissue, no gap and no pathomorphological changes were observed; (**b**) X-ray after treatment with biocement C; no signs of tissue irritation were observed; (**c**) untreated defect; new bone formation is absent; (**d**) MRI, treatment with biocement CX; the treated defects were completely filled with the newly formed cartilage to the expected level equal to the adjacent cartilage, and tissue surface was smooth, well-defined. (**e**) MRI, treatment with biocement C; newly formed cartilage had a comparable thickness to the adjacent tissue, slightly hyperintense areas were visible; (**f**) MRI of the untreated defect; the defect in its full extent shows hyperintense and hypointense signal alterations; (**g**) CT image, treatment with CX, partial degradation of biocement, unresorbed part of biocement was visible under the subchondral bone, the surface of biocement was covered with new bone tissue; (**h**) CT image, treatment with C, biocement was not completely degraded and resorbed, and integration of the biocement with the adjacent bone was confirmed; (**i**) untreated defect, the osteochondral defect was not completely covered by the newly formed bone, and a large cavity remains inside the bone; arrows: indicated areas of the healed defect.

Radiological examination confirmed that the process of biodegradation of biocements was incomplete, and biomaterials were only partially resorbed. Unresorbed biomaterials appeared radiodense on both X-ray and CT images (Figure 5). Remnants of both biocement systems were present in the trabecular bone. The subchondral bone was intact. The area around the defect was homogeneous, without pathological irritation, and without osteolytic changes. The new bone was attached directly to the surface of the material, with no free spaces, gaps, or fibrous capsules between them. No swelling of the adjacent soft parts was present. We observed areas of less echogenic matter which verifies primarily the newly formed bone tissue. CT findings indicate good healing of the defects without any signs of irritation (Figure 5).

X-ray images did not indicate the presence of pathological changes, like narrowing of the joint space, the formation of subchondral cysts, osteonecrosis, or osteophytosis in the investigated groups. Similarly, there were no signs of osteoarthritis. In most cases, the new bone tissue in the untreated defects was only partially visible and was formed closer to the bone surface up to a maximum thickness of 2.5 mm, but the bone defects were not completely filled by newly formed bone. In a few cases, several defects were without any new trabecular bone formation.

### 2.4. Magnetic Resonance Imaging

MRI results are consistent with previous evaluations. In the CX group, the treated osteochondral defects were filled with tissue that had a well-defined surface with fine surface irregularities. The filling of the defects was at the expected level consistent with the surrounding healthy articular cartilage. The thickness of the newly formed tissue was comparable to the adjacent cartilage. The structure of the regenerated cartilage was mainly homogeneous with an isointense signal and showed no cracks and fissures. Areas with a hypointense or hyperintense signal were rarely observed. The interface between the site of the original defect and the healthy cartilage was not visible. According to the Mocart 2.0 Knee score, significant differences in the total score were seen between osteochondral defects treated with biocement CX and untreated defects (CX and untreated defect *p* < 0.05).

**Scheme 3:**
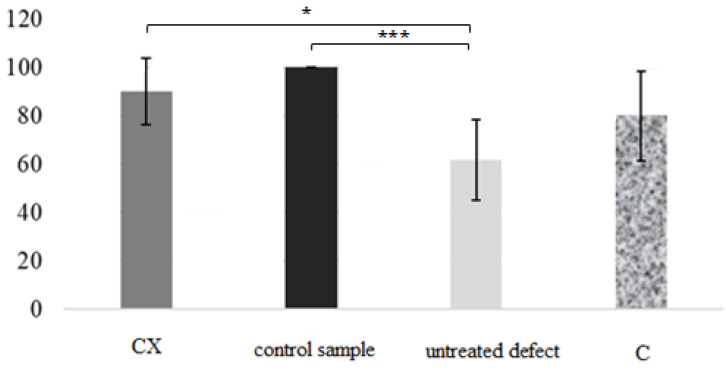
Results of MOCART 2.0 knee score. CX—osteochondral defects treated with biocement CX, control samples—healthy native cartilage obtained from the osteochondral cylinders extracted by OATS kits; untreated defects—empty defects left to heal spontaneously. C—osteochondral defects treated with biocement C. * = *p* < 0.05, *** = *p* < 0.001.

Osteochondral defects treated with biocement C showed similar results with greater surface irregularity and the presence of hypointense sections in the newly formed tissue. Adjacent areas were without signs of perifocal reactive bone oedema, only in two cases we found a border of residual bone oedema in the group treated with biomaterial C. In the Mocart 2.0 Knee score, higher values were obtained for defects treated with CX, but the differences between C and CX were not significant (Scheme 3).

Untreated defects were in most cases incompletely filled with inhomogeneous tissue. Insufficient filling of the defect or hypertrophy was visible, resulting in an uneven joint contour. Areas with hypointense or hyperintense signals were also visible. The differences between the untreated defects compared to the control sample were statistically significant (*p* < 0.001).

We noted different signal alterations in trabecular bone in defects treated with biomaterials, indicating incomplete biocement resorption and an ongoing process of bone remodelling with typical signs of healing. The used biomaterials CX and C did not evoke specific degenerative changes in the joint cavity nor in the surrounding native cartilage or bone. Synovitis was not noted in any of the scanned sheep.

## 3. Discussion

Our study aimed to observe the effect of phytic acid–phytase hardening liquid added to tetracalcium phosphate–monetit on the improvement of healing of osteochondral defects. The addition of phytic acid–phytase complex compared to biocement with classic fast-setting cement systems positively influenced the healing outcome with a higher overall score achieved in macroscopic, histologic, and MRI scoring systems.

We were based on the fact that CaPs have been widely studied for the regeneration of bone tissue in various forms, such as different types of biocements, coatings, scaffolds, and others [8,34]. At the same time, many studies have been conducted to improve the efficiency of calcium phosphate biomaterials in combination with diverse therapeutic agents. Phytic acid is a naturally extracted substance from plants that has excellent biocompatibility, and anti-inflammatory, antioxidant, and antibacterial effects [35]. Anticancer activity and antiosteoporotic properties were also described [36,37]. The phytic acid acted as a phosphate source and setting modifier, as well as the cement chemical composition modifier [33,38]. Numerous in vitro studies confirmed the positive influence of phytic acid on osteogenic activity, cell viability, and proliferation.

Cytocompatibility assessed by in vitro assays using osteoblastic and osteoclastic cells showed better results for sodium pyrophosphate and phytic acid with a triple rise in metabolic activity of the cells, while samples containing, for example, citric acid, showed reduced cell number and cellular metabolic activity. Phytic acid also showed no inhibitory effect on osteoclast activity and proliferation. The authors of the study reported that phytic acid, as a new setting retardant for dicalcium phosphate cements, appears promising concerning material properties and biological behaviour [38].

In the in vitro study by Zhang et al. [39], Mg ions incorporated into the phytic acid and zoledronic acid of a metal–organic complex coating was built on the Mg substrate. The proliferation of preosteoblast cells (MC3T3-E1) was promoted due to the well-controlled release of Mg ions and the osteocompatible phytic acid molecule. This metal–organic complex coating offers a promising modification for Mg-based orthopedic implants in the framework of osteoporotic fracture healing [39].

Liu et al. [19] used a mixed solution of phytic acid and Ca(OH)_2_, as coatings on the titanium surface. This modified surface has excellent surface properties and can stimulate the osteogenic differentiation of MSC, cell adhesion, and proliferation.

Evaluation of the osteogenic ability of phytate in vitro was performed using hMSC cells in the study by Asensio et al. [18]. The results obtained in this study contribute to the understanding of the role of phytic acid and phytic acid complexes bearing bioactive cations Zn2+ and Sr2+ (SrPhy and ZnPhy) in the differentiation of mesenchymal stem cells into osteoblasts. The phytate compounds used were not cytotoxic. Individual doses of phytate complexes increased the cytocompatibility, and they were able to influence the osteogenic differentiation of mesenchymal stem cells to the osteoblastic phenotype in terms of ALP activity and the mineralization of the matrix. The results demonstrated that phytic acid and the phytate complexes showed suitable in vitro properties and are appropriate as osteoinductive factors for biomedical applications in bone regenerative therapies [18].

The cytocompatibility of the novel biocement CX used in our study was verified in an in vitro study on preosteoblastic MC3T3E1 cells and the noncytotoxic character of the cement, as well as good viability, proliferation, and ALP activity of osteoblasts, were observed [30].

In general, the composition of biomaterials, which contain calcium and phosphate, shows good cytocompatibility and improves contact with bone as well as new bone growth. Biocompatibility is a very important factor in the application of any biomaterial to the medical field [40]. In the current study, a clinically relevant sheep model was used to observe the biocompatibility of the CaP-based material with the phytic acid–phytase complex. The in vivo results of our study indicate the excellent biocompatibility of tetracalcium phosphate–monetit cement with phytic acid–phytase hardening liquid. No inflammatory reaction, signs of infection, tissue irritation, or pathomorphological changes were observed in the treated groups. The addition of phytic did not negatively affect the compatibility of the biocement CX with the surrounding tissue.

The expected immune response after the application of biomaterials inhibits fibrous encapsulation and provides a suitable osteogenic environment for bone regeneration [41]. An unwanted immune response causes chronic inflammation around the implants leading to the formation of a fibrous capsule isolating the implant from the adjacent tissue, negatively affects the adhesion of cells on the surface of the used material, and can lead to the failure of the implantation process [42]. In this in vivo study, no fibrous encapsulation around newly formed tissue and unresorbed biocements was observed after 6 months of treatment. The unresorbed biocements in trabecular bones were firmly and strongly connected to the adjacent tissue and showed excellent osteointegration. Moreover, no gaps were observed between the material and the host bone and cartilage tissue.

According to Dong et al. [43], osteointegration can be improved by modifying the implant surface. In the study, a titanium surface was enhanced with a mixture of phytic acid and calcium hydroxide. In vivo, the evaluation of osteogenesis in a diabetic rat model revealed that a Ca-phytic acid coating improved the peri-implant osteointegration.

The effect of phosphoserine/tetracalcium phosphate cement was evaluated in vivo in the preclinical study by Bingol et al. [44]. No evidence of cytotoxicity or abnormal effects on surrounding tissues was observed, and excellent osteointegration and osteoconduction were noticed after implantation of the biocement into critical defects created in the distal region of the rabbit femur. After four months, the modified cement was fully replaced by bone, unlike the controls, which showed the presence of either isolated particles or soft tissue.

In the current study, the biocements were not completely degraded and replaced by new trabecular bone, which can be seen on CT and X-ray images and also macroscopically after the removal of the osteochondral cylinders. Contrary to trabecular bone, biocement residues were not observed in the newly formed cartilage and subchondral bone.

According to Sahin et al. [45], an appropriate rate of resorption is a very important parameter that may vary depending on clinical applications, age, anatomic position, or general metabolic health. The degradation and resorption can take from 3 to 36 months before the material is fully replaced by bone. Although the insufficient degradability of the material in trabecular bone was observed in our study, it was associated with the continuous formation of subchondral bone and cartilage with the presence of hyaline cartilage layers, although fine fibres were noted in the cartilage matrix.

The study by Guo et al. [29] described a new calcium phosphate-like material with added phytic acid that can speed up the degradation process of bone substitutes. In contrast to this study, we did not observe an acceleration of the degradation process of biocement CX compared to the degradation process of biocement C, which was without the addition of phytic acid. In the aforementioned in vivo study by Guo et al. [29], hydroxyapatite (HA), β-tricalcium phosphate (β-TCP), and calcium phytates were applied to femoral condyle defects in rats. The study reported that calcium phytates showed excellent osteogenic properties compared to HA and β-TCP and indicates that they are a promising candidate for bone-defect repair [29].

CaPs-based biomaterials are usually used in the regeneration of bone tissue. There are few studies in the available literature that deal with the effect of CaP on cartilage tissue. The findings of several studies confirm that calcium phosphates promote chondrocyte proliferation and biosynthesis [46,47,48], although the mechanisms of this effect remain unclear. An in vitro study by Boushell et al. [46] stated that a higher Ca/P ratio of 1.41 led to increased chondrocyte proliferation and proteoglycan production. It was also reported that calcium concentration plays an important role in regulating enzyme activity in chondrocytes [49]. 

The results of our study confirm the positive impact of CAPs on articular cartilage and subchondral bone healing. The results obtained were significantly better for treated defects than untreated defects in all scoring systems. Although the differences between osteochondral defects treated with CX and C were not significant in the scoring systems, higher values in scores were obtained in the overall macroscopic appearance and histological evaluation, as well as the MRI data in the defects treated with biocement CX.

Testing the suitability of β-TCP ceramics for the repair of osteochondral defects in the sheep animal model was carried out by the study Bernstein et al. [50]. Macroscopically, no signs of inflammation or infection were observed. Cartilage formation began at the outer edge and continued to the centre. Although the O’Driscoll score values corresponding to natural cartilage were not reached after 1 year, hyaline cartilage was found in the samples and very good integration of the newly formed cartilage into the surrounding native cartilage was observed, which is consistent with our results. Treated defects had less than 25% of cells in clusters, whereas untreated defects had more than half of the cells involved in clusters. In our study, larger cluster formation of chondrocytes was observed in untreated defects and in treated defects mainly at the transition between healthy and newly formed cartilage.

Considering that there is still no ideal biomaterial for the treatment of osteochondral defects, there is still great interest in new treatment options. We were interested in contributing to this area of research by using a new, originally prepared biomaterial CX with the innovative composition of tetracalcium phosphate–monetite and phytic acid–phytase as the hardening liquid, which, to our knowledge, has not yet been used and evaluated in vivo. Phytic acid has excellent biological properties, and its addition had an evident effect on the treatment of osteochondral defects. We have to state, however, that, even though a higher score was achieved in the implemented scoring systems for biocement CX compared to biocement C without phytic acid, the differences between the treated groups were not significant. Nevertheless, we consider CX biocement to be a suitable biomaterial for the effective treatment of osteochondral defects in sheep, which induces the formation of cartilaginous and bone tissue.

There may be some potential limitations in this study. The first is the insufficient immobilization of the limb after the surgical intervention. The animals after surgery were returned to their cages, without the possibility of offloading the limb, as is standard for human patients after surgery. The second limitation concerns biodegradation. Although new tissue was formed after 6 months, this period of time was not sufficient for the complete degradation of the biocements. It would be advisable to monitor the biodegradation process after a longer period of time, e.g., 1–2 years, and similarly observe the nature of the newly formed tissue. Radiological examination and MRI were performed immediately after the euthanasia of the animals. It would be worthwhile to do the radiological examination during life and monitor the individual stages of healing. For technical reasons, it was not implemented in this study.

## 4. Materials and Methods

### 4.1. Biocement C and CX Preparation

Biocement C and biocement CX were prepared according to Medvecky et al. [30] and Medvecky et al. [33]. Briefly, the tetracalcium phosphate Ca_4_(PO_4_)_2_O was prepared by milling of solid-state reaction product after the annealing of an equimolar mixture of dicalcium phosphate anhydrous CaHPO (Fluka, Buchs, Switzerland) and calcium carbonate CaCO_3_ (Sigma-Aldrich, Saint Louis, MO, USA) at 1450 °C for 5 h in a planetary ball mill (Fritsch, Idar-Oberstein, Germany, 730 rpm) for 2 h.

The C biocement composed of a tetracalcium phosphate–monetite powder mixture was prepared by an in situ reaction of tetracalcium phosphate and orthophosphoric acid (H_3_PO_4_, 86%, Merc, Darmstadt, Germany) in 80% ethanol by using a planetary ball mill at 730 rpm for 30 min. The final Ca/P mole ratio was 1.67 (stoichiometric HA) and the cement paste was prepared by mixing the powder mixtures with 2%NaH_2_PO_4_ (as a liquid component) at a P/L ratio = 2.

**Figure 6:**
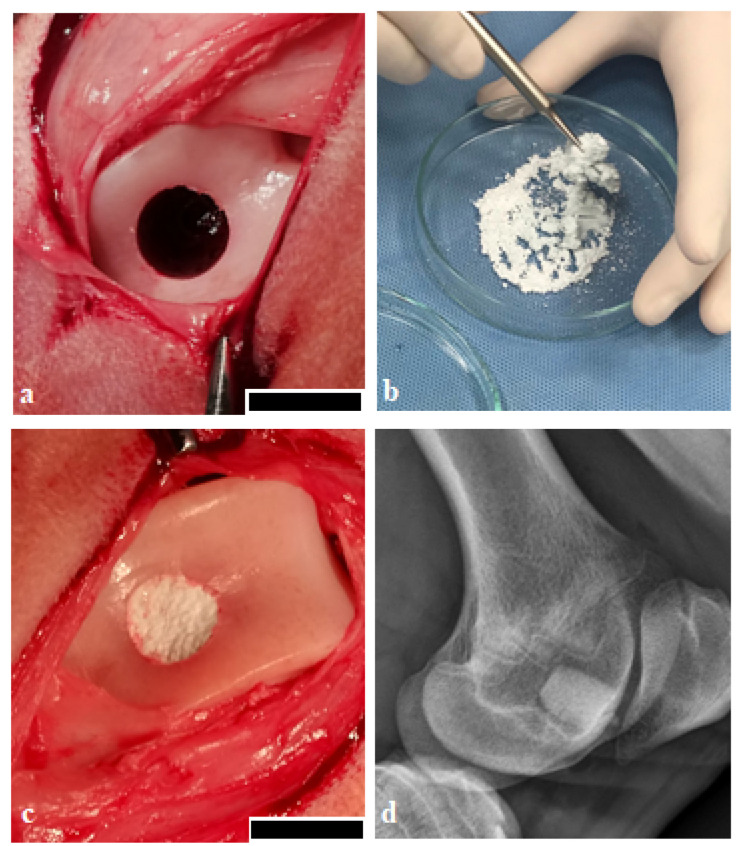
Surgical procedure. (**a**) osteochondral defect of 8 mm diameter and 10 mm depth was created using an OATS kit in the trochlea ossis femoris, scale bar 1 cm, (**b**) preparation of the final biocement paste (biocement CX) before application to the osteochondral defect by mixing the powder and liquid components, (**c**) osteochondral defect after filling with biocement paste, scale bar 1 cm, and (**d**) X-ray confirmation after surgery.

In the case of enzymatically hardened CX biocement, the phytic acid (content 8 wt%) and phytase (Naturphos^®^, BASF, Ludwigshafen, Germany) were admixed to a liquid component. Phytic acid and phytase were separately dissolved in a solution of 0.5% acetic acid, and the enzyme reaction was in progress for 30 s. The final molar ratio of Ca/P (including phosphates from phytic acid) was 1.50. Hot-air sterilization at a temperature of 160 °C/90 min was used to sterilize the powder mixture. The final cement paste was prepared just before application to the osteochondral defect by mixing the powder mixtures with the liquid components (Figure 6).

### 4.2. Animals, Surgical Procedure, Postsurgical Period

This study was performed on 18 healthy female sheep of the Valachian/Merino sheep breed. The ethical approval for animal procedures was obtained from the State Veterinary and Food Administration of the Slovak Republic (No. 2220/17-221). At the time of the surgical intervention, animals were from 2 to 3 years old and from 62 to 73 kg in weight. Anaesthesia of the animals was induced with butorphanol (0.1 mg/kg, Butomidor 10 mg/mL, Richter Pharma, Wels, Austria), and medetomidine 0.02 mg/kg (Cepetor 1 mg/mL, CP-Pharma Handelsgesellschaft, GmbH, Burgdorf, Germany) applied intramuscularly, and ketamine 8 mg/kg (Ketamidor 100 mg/mL, Richter Pharma, Wels, Austria) was applied intravenously.

All surgical procedures were performed under aseptic conditions. In general anaesthesia, the left hind limb of all animals was washed and shaved around the knee joint and prepared with a Betadine and alcohol solution using a sterile technique. A 10 cm-long surgical incision was made through the skin and soft tissue at the medial border of the patella. After luxation of the patella, the trochlea femoris was exposed. Osteochondral defects were created using a standard osteochondral autograft transfer system (OATS; Arthrex, Naples, FL, USA) 8 mm in diameter and 10 mm deep in the trochlea ossis femoris (Figure 6). Animals were divided into 3 groups. In group 1 (*n* = 6), osteochondral defects were treated with the biocement CX, in group 2 (*n* = 6) osteochondral defects were treated with the biocement C, and in group 3, untreated defects (*n* = 6) and empty defects were left to heal spontaneously. Control samples (*n* = 6) were taken from the healthy native cartilage obtained from extracted osteochondral cylinders. Immediately after filling the osteochondral defect, the skin and soft tissues were sutured, and the surgical wound was sprayed with an aluminium fluid spray. The absence of bone fractures, possible arthropathy, and the correct orientation of the osteochondral defects were confirmed by X-ray (Philips Digital Diagnost, Delft, The Netherlands) and the surgical wound was finally sprayed with aluminium spray.

After the surgical intervention, the animals were returned to cages and allowed to move freely without external fixation and support. The antibiotic prophylaxis, oxytetracycline dihydricum 20 mg/kg (Alamycin LA a.u.v., Norbrook, Newry, UK), was applied intramuscularly for 7 days after surgery every second day. Flunixin meglumine 2.2 mg/kg (Flunixin a.u.v., Norbrook, Newry, UK) was administered intramuscularly once daily for pain control for 7 days. The sheep were euthanized 6 months after surgery using Xylazine 0.2 mg/kg (Rometar 20 mg/mL inj., Bioveta, Nitra, Slovak Republic) applied intramuscularly, and Ketamin 2 mg kg (Narkamon 100mg/mL inj., Bioveta, Nitra, Slovak Republic) administered intravenously. The knee joints were investigated by X-ray, computed tomography, and magnetic resonance imaging, and then opened and evaluated for macroscopic appearance and histological analysis.

### 4.3. Macroscopic Analysis

A semiquantitative macroscopic scoring system according to Goebel et al. [51] was used for the macroscopic evaluation of the articular cartilage, and the description of cartilage repair. It ranges from 0 points, indicating excellent repair of articular cartilage, to 20 points, indicating cartilage defect without any repair tissue. Five main parameters for cartilage assessment with 25 items were formulated as follows.

**I**. Color of the repair tissue: hyaline or white (0), predominantly white (>50%) (1), predominantly translucent (>50%) (2), translucent (3), no repair tissue (4). **II.** presence of blood vessels in the repair tissue: no (0), less than 25% of the repair tissue (1), 25–50% of the repair tissue (2), 50–75% of the repair tissue (3), more than 75% of the repair tissue (4). **III**. surface of the repair tissue: smooth, homogeneous (0), smooth, heterogeneous (1), fibrillated (2), incomplete new repair tissue (3), no repair tissue (4). **IV**. filling of the defect: in level with adjacent cartilage (0), >50% repair of defect depth or hypertrophy (1), 0% repair of defect depth (2), subchondral bone damage (3), no repair tissue (4). **V**. degeneration of adjacent articular cartilage: normal (0), cracks and/or fibrillations in integration zone (1), diffuse osteoarthritic changes (2), extension of the defect into the adjacent cartilage (3), subchondral bone damage (4).

For the worst possible result, a total of 20 points is achieved [51].

### 4.4. Histologic Analysis

Neoformed tissue samples for histologic analysis were obtained using the Osteochondral Autograft Transfer System (OATS, Arthtrex, Naples, FL, USA). Osteochondral cylinders were first fixed in neutral formalin (4%), decalcified in chelaton, then dehydrated in an ethanol series, and, finally, inserted into paraffin. Histological sections were made at 7 µm using a Leica microtome (Leica, Bensheim, Germany). Subsequently, histological sections were stained according to standard protocols with hematoxylin–eosin staining to evaluate the new tissue structure and chondrocyte morphology, Safranin O and Alcian blue staining to determine proteoglycan presence and distribution in the extracellular matrix. Picrosirius red and Masson’s trichrome staining were used to demonstrate the presence of collagen. Histological samples were examined with an Olympus CX 43 light microscope (Olympus Corporation, Tokyo, Japan), and a 300MIPromicam digital camera (Promicra, Prague, Czech Republic). For each osteochondral defect, two observers evaluated five histological specimens from each staining.

Histological analysis was performed using a modified O’Driscoll histological scoring system [52]. The maximum number of points is 28 points, which means the best result. Number of points in individual categories according to modified O’Driscoll score: 

**I**. Nature of predominant tissue: hyaline cartilage (4), mostly hyaline cartilage (3), mixed hyaline and fibrocartilage (2), mostly fibrocartilage (1), some fibrocartilage, mostly nonchondrocytic cells (0); **II**. structural characteristics: (A) surface irregularity: smooth and intact (3), superficial horizontal lamination (2), fissures (1), severe disruption, including fibrillation (0); (B) structural integrity, homogeneity: normal (2), slight disruption, including cyst (1), severe disintegration, disruptions (0); (C) thickness: 100% of normal adjacent cartilage (2), 50–100%or thicker than normal (1), 0–50% of normal cartilage (0); (D) bonding to adjacent cartilage: bonded at both ends of graft (2), bonded at one end or partially both ends (1), not bonded (0); **III.** freedom from cellular ranges of degeneration: (A) hypocellularity: normal cellularity (2), slight hypocellularity (1), moderate hypocellularity or hypercellularity (0); (B) chondrocyte clustering: no clusters (2), <25% of the cells (1), 25–100% of the cells (0); **IV.** freedom from degenerate ranges in adjacent cartilage: normal cellularity, no clusters, normal staining (3), normal cellularity, mild clusters, moderate staining (2), mild or moderate hypo/hypercellularity, slight staining (1), severe hypocellularity, poor or no staining (0); **V**. subchondral bone: (A) reconstruction of subchondral bone: normal (3), reduced subchondral bone reconstruction (2), minimal subchondral bone reconstruction (1), no subchondral bone reconstruction (0); (B) inflammatory response in subchondral bone region: none/mild (2), moderate (1), severe (0); **VI.** Safranin O staining: normal or near normal (3), moderate (2), slight (1), none (0).

### 4.5. X-ray, CT, MRI

X-ray analysis was performed by X-ray equipment (Philips Digital Diagnost, Delft, The Netherlands) at 55–60 kV; 1.8–4.9 mAs; and the pixel size at 0.133 mm. X-ray examination was used to evaluate potential osteoarthritis and for the assessment of subchondral cystic masses or osteophytosis.

Computed tomography analysis was made with a CT (Philips Brilliance 40-slice CT, Delft, The Netherlands) with the following parameters: 120 kV, 250 mA, 300 mAs, pixel size 0.283 mm, and the thickness of slices 2 mm. The presence of nondegraded biocement as well as potential pathological and inflammatory changes were evaluated. 

Magnetic resonance imaging was performed by an MR-imaging system at 1.2 T (Hitachi Oasis, Open system, Hitachi Medical Systems Holding AG, Tokyo, Japan). The following scanning parameters were used: ET:10; TR: 3500–3805; TE:36.0; pixel size: 0.166. Characteristics of the hyaline cartilage, defect filling, repair-tissue surface, possible chondral lesions, irregularities in the neoformed surface, and subchondral changes were assessed using the MOCART (magnetic resonance observation of cartilage repair tissue) 2.0 knee score developed by Schreiner et al. [3]. Total score ranges were from 0 to 100 points with 100 points being the best result.

These parameters were monitored: **I**. volume fill of cartilage defect: 1. complete filling or minor hypertrophy: 100% to 150% filling of total defect volume (20), 2. major hypertrophy ≥ 150% or 75% to 99% filling of total defect volume (15), 3. 50% to 74% filling of total defect volume (10), 4. 25% to 49% filling of total defect volume (5), 5. <25% filling of total defect volume or complete delamination in situ (0). **II**. integration into adjacent cartilage: 1. complete integration (15), 2. split-like defect at repair tissue and native cartilage interface ≤ 2 mm (10), 3. defect at repair tissue and native cartilage interface >2 mm, but <0% of repair-tissue length (5), defect at repair tissue and native cartilage interface ≥50% of repair-tissue length (0). **III.** surface of the repair tissue: surface of the repair tissue (10), surface irregular <50% of repair-tissue diameter (5), surface irregular ≥50% of repair-tissue diameter (0). **IV**. Structure of the repair tissue: homogeneous (10), inhomogeneous (0).**V.** signal intensity of the repair tissue: Normal (15), minor abnormal—minor hyperintense or minor hypointense (10), Severely abnormal—almost fluid-like or close to subchondral plate signal (0). **VI.** bony defect or bony overgrowth: no bony defect or bony overgrowth (10), bony defect: depth < thickness of adjacent cartilage or overgrowth <50% of adjacent cartilage (5), bony defect: depth ≥ thickness of adjacent cartilage or overgrowth ≥50% of adjacent cartilage (0). **VII**. subchondral changes: no major subchondral changes (20), minor oedema-like marrow signal—maximum diameter < 50% of repair-tissue diameter (15), severe oedema-like marrow signal—maximum diameter ≥ 50% of repair-tissue diameter (10), subchondral cyst ≥ 5 mm in longest diameter or osteonecrosis-like signal (0).

### 4.6. Statistical Analysis

Differences between groups were assessed by an unpaired *t*-test and one-way ANOVA with Tukey’s post hoc analysis (GraphPad Prism 7.0 for Windows, GraphPad Software, San Diego, CA, USA). The results were reported as means ± standard deviations of means (SD). Differences were considered statistically significant at the levels of * = *p* < 0.05; ** = *p* < 0.01; and *** = *p* < 0.001.

## 5. Conclusions

We can conclude that the hardening liquid composed of phytic acid–phytase added to tetracalciumphosphate–monetit had a positive effect on the healing of osteochondral defects. The novel biocement stimulated the formation of bone tissue and cartilage with a predominant structure of hyaline cartilage. The evaluation of all scoring systems was better for CX, although the differences in total scores between the biocement C and CX groups were not significant. The results of this preclinical study demonstrated the excellent biocompatibility and regenerative potential of biocement CX, which may be interesting material for further research and use in human medicine.

## Figures and Tables

**Table 1:** Total points in scoring systems (mean ± SD).

Scoring Systems	CX	Control Sample	Untreated Defect	C
Modified O’Driscoll scoring system	21.2 ± 1.3	27.2 ± 1.3	15.6 ± 1.5	20.8 ± 1.6
Macroscopic semiquantitative scoring system	5.6 ± 1.7	0	14.4 ± 2.1	7.2 ± 1.3
MOCART 2.0 Knee score	90 ± 13.8	100	61.6 ± 16.6	80 ± 18.7

## Data Availability

Data are contained within the article.
